# *In vitro* and *in vivo* correlation of injectable lipid-based nanomedicines: From dissolution to protein coronas

**DOI:** 10.1016/j.mtbio.2025.102393

**Published:** 2025-10-08

**Authors:** Huan Wang, Xiying Wu, Junji Wang, Xiaoyi Wang, Ying Lu

**Affiliations:** a Department of Pharmaceutical Sciences, School of Pharmacy, Naval Medical University, Shanghai, China; b Shanghai Skin Disease Hospital, School of Medicine Tongji University, Shanghai, China; c State Key Laboratory of Biopharmaceutical Preparation and Delivery, Institute of Process Engineering, Chinese Academy of Sciences, Beijing, China

**Keywords:** Lipid-based nanomedicines, *In vitro-in vivo* correlation, Protein coronas, Dissolution, *In vivo* delivery process

## Abstract

Lipid-based nanomedicines (LBNMs) represent a transformative frontier in improving drug bioavailability and achieving spatiotemporally precise delivery of therapeutic agents. However, establishing robust *in vitro-in vivo* correlation (IVIVC) for LBNMs remains a significant challenge due to the complexity of their interactions with biological systems after administration. Conventional IVIVC approaches, often relying on simplistic *in vitro* dissolution models, frequently fail to recapitulate dynamic physiological processes, leading to discrepancies between predicted and actual *in vivo* performance. Recent advancements in understanding protein coronas-mediated phenomena have revolutionized nanomedicines research by unraveling how adsorbed biomolecules at the nanoparticle interface dictate physiological processes such as immune system interplay and biodistribution. This review systematically evaluates the state-of-the-art models for IVIVC of LBNMs and the emerging roles of protein coronas in bridging the gap between *in vitro* characterization and *in vivo* behavior, emphasizing the necessity of integrating protein coronas analysis into IVIVC frameworks.

## Highlights

• Conventional dissolution-focused IVIVC models fail to predict *in vivo* behavior of injectable complex LBNMs.
• Protein coronas dynamically reshape LBNM’ biological identity and biodistribution, bridging IVIVC gaps of LBNMs.
• Establishing correlations between physicochemical properties, key PC composition, and in vivo performance prioritizes the development of IVIVC frameworks of LBNMs.

## Graphical abstract

**Figure:**
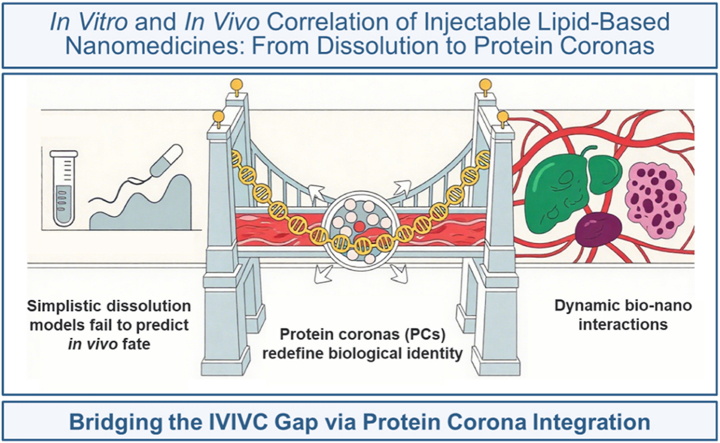

## 1 Introduction

Nanomedicines possess the capability to encapsulate diverse therapeutics, affording advantages such as improved drug solubility, prolonged circulation time, controlled and sustained drug release, as well as targeted delivery [1,2]. In recent decades, the advent of lipid-based nanomedicines (LBNMs) delivery systems has propelled the realm of nanomedicines to unprecedented heights. Distinguished from other nanocarriers, LBNMs boast superior biocompatibility, good biodegradability and minimal immunogenicity, yielding great potential for clinical applications including cancer, viral or fungal infections, analgesia, and gene delivery [[3], [4], [5]]. However, the clinical translation rate of LBNMs remains disappointing considering significant investments in research [6]. In addition, no clinically approved varieties exist for “smart nanomedicines”, such as active targeted or responsive nanomedicines. Notably, the approved LBNMs often fail to meet expected therapeutic outcomes or present new side effects with unclear underlying mechanisms [7].

Despite the plethora of reports on innovative nanomaterials, nanocarriers, nanostructures, and nano-delivery methodologies in recent years, the *in vivo* delivery process of LBNMs remains largely untapped and enigmatic [8]. Critical aspects such as blood circulation, tissue penetration, biodistribution, interactions with target and non-target cells, and intracellular trafficking are still understudied, creating a significant knowledge gap in the clinical translation of these therapeutics [9]. The intricate *in vivo* processes of LBNMs often lead to substantial discrepancies between the anticipated outcomes based on *in vitro* designs and the actual performance observed *in vivo*. Understanding and establishing the correlation between the physicochemical properties of LBNMs and their biological interactions poses a significant challenge in the field of nanotherapeutics. Clarifying the *in vivo* delivery process and revealing the key regulatory mechanisms are important links to facilitate new LBNMs development and clinical translation.

The concept of *in vitro*-*in vivo* correlation (IVIVC) refers to the quantitative relationship between the physicochemical properties of a drug and its biological behavior. Conventionally, the evaluation of IVIVC in nanomedicines has relied on *in vitro* dissolution testing, entailing sophisticated mathematical modeling of dissolution data and correlating it with plasma drug concentration-time profiles [10,11]. This methodology facilitates the expedited screening of pharmaceutical formulations and serves as a valuable reference for the establishment of bioequivalence studies. However, *in vitro* dissolution studies are insufficient for evaluating LBNMs with slow drug release or those where drug premature release is undesirable (e.g., nucleic acid-encapsulating formulations). More critically, conventional IVIVC methods overlook the bioactivity of LBNMs themselves. This limitation is particularly pronounced for injectable LBNMs administered into the bloodstream, where the intrinsic biological activity of the LBNMs cannot be ignored. Recent studies have revealed that the protein coronas (PCs), a layer of biomolecular adsorption surrounding LBNMs, play pivotal roles in cellular interactions and uptake, ultimately dominating the *in vivo* behavior of LBNMs [[12], [13], [14]]. Therefore, the integration of *in vitro* dissolution with the PCs constitutes a fundamental framework for elucidating the IVIVC of LBNMs, which is essential for their successful clinical translation.

This review primarily summarizes the research progress regarding the IVIVC of LBNMs, organized into five sections. Initially, we outline the key milestones achieved in the study of LBNMs. Subsequently, we highlight the emerging challenges associated with the efficient *in vivo* delivery of LBNMs and call for a rational comprehension of the intricate *in vivo* processes. Then, we introduce the state-of-the-art models for IVIVC of LBNMs. Finally, we elaborate on the progress in IVIVC of LBNMs from the perspectives of *in vitro* dissolution and *in vivo* PCs analysis, respectively. This review focuses on the *in vivo* delivery process, delving into the interactions between PCs and LBNMs, as well as their contributions to the development of IVIVC, with the ultimate goal of advancing LBNMs from the laboratory to the bedside.

## 2 General aspects of LBNMs

**Fig. 1:**
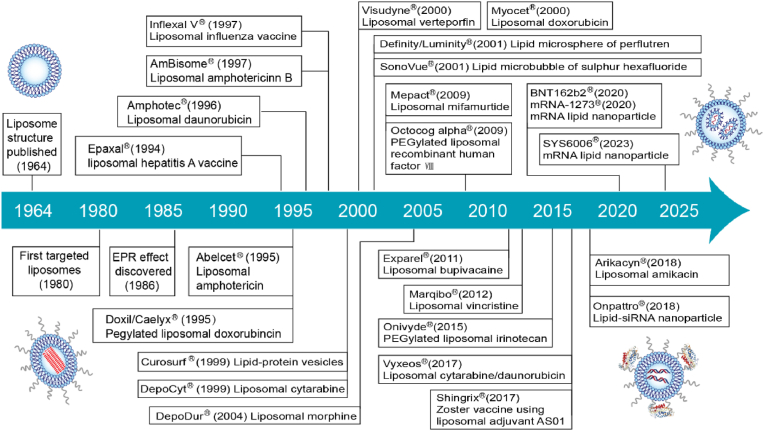
The milestones of injectable complex LBNMs. Represented by typical examples like Doxil® and mRNA LNP vaccines (mRNA-1273 and BNT162b2), LBNMs exhibit considerable potential in optimizing drug formulations, mitigating drug toxicity, and expanding therapeutic application scopes.

More than 60 years have passed since Alec Bangham and his colleagues first discovered the ‘spontaneous formation of closed vesicles from phospholipids’ in 1961 [15,16]. LBNMs have already witted great success in basic research, clinical or commercial products. According to their composition and structure, LBNMs can be classified into at least six different types: *i.e.*, liposomes, lipid nanoparticles (LNPs), lipid nanodiscs (LNDs), solid lipid nanoparticles (SLNs), and nanostructured lipid carriers (NLCs) and lipid nanoemulsions (LNEs). The structures and the milestones of LBNMs are shown in Fig. 1.

### 2.1 Liposomes

Liposomes are the oldest and the most common type of LBNMs. They are spherical vesicles with enclosed central aqueous chambers formed by single or concentric multiple double layers of lipid self-assembly with particle sizes ranging from 30 nm to microns. Liposomes exhibit versatility in drug encapsulation, enabling them to accommodate both hydrophobic drugs within the lipid bilayer and hydrophilic drugs within the aqueous core. Some cationic liposomes possess the capability to electrostatically bind anionic nucleic acids to the surface. Additionally, by strategically incorporating additional functional lipids onto their surface, liposomes can be endowed with diverse functionalities, such as extended circulation time and active targeting capabilities.

As drug carriers, liposomes can protect the encapsulated active ingredients from degradation, prolong the half-life, achieve controlled release, and selectively deliver the drugs to the site of pathogenesis, thereby reducing systemic side effects, increasing the maximum tolerated dose, and improving efficacy. For instance, in the realm of anti-tumor drug carriers, doxorubicin, irinotecan, vincristine and cisplatin liposomes have achieved international commercialization. Mitoxantrone liposomes are actively undergoing several clinical trials (NCT05522192, NCT05941585, NCT05875428, NCT06447090, *etc*.). Thermosensitive liposomal doxorubicin was determined for treatment of non-resectable hepatocellular carcinoma (NCT00617981). Meanwhile, liposomes encapsulating nucleic acid drug are also advancing through various clinical trials (e.g., cationic liposome-DNA complexes for relapsed or refractory leukemia (NCT00860522), liposomal mRNA vaccines for ovarian cancer (NCT04163094), highlighting the immense promise of liposomes in cancer therapy. In anti-parasite drug carriers, thiabendazole liposomes and albendazole liposomes are utilized to boost drug bioavailability, reduce dosage, and mitigate toxic and adverse effects [17]. In the realm of antibacterial drug carriers, gentamicin liposomes and amphotericin B liposomes have shown potential in reducing drug resistance and diminishing cardiotoxicity [[17], [18], [19]].

### 2.2 Lipid nanoparticles

Lipid nanoparticles (LNPs) are spherical nanoparticles composed of cationic/ionizable lipids, amphiphilic phospholipids, cholesterol, and polyethylene glycol (PEG)-modified lipids. LNPs are one of the most successful LBNMs for delivery of nucleic acid drugs, in which ionizable lipids can be converted into cationic lipids for encapsulation of nucleic acid drugs through electrostatic interactions [20,21]. Unlike liposomes, LNPs do not possess a closed lipid bilayer structure with an internal aqueous core. The most widely accepted structural of LNPs is an inner nucleic acid drugs and lipids form reverse micelle nuclei surrounded by outer lipids. LNPs can protect the nucleic acid cargos from degradation and efficiently introduce them into the cytoplasm [22]. In addition, LNPs can also function as adjuvants to amplify the immune response and vaccinated by different routes such as injection or inhalation [23].

The LNPs-RNA complexes are revolutionizing the medicine. In 2018, the first FDA-approved Onpattro® (patisiran®), effectively delivered small interfering RNA (siRNA) to the liver for the treatment of transthyretin-mediated amyloidosis [24]. In 2020, COVID-19 mRNA vaccines (mRNA-1273 by Moderna and BNT162b2 by Pfizer) received FDA Emergency Use Authorization, marking a milestone for mRNA-LNPs [25]. In addition to the application as prophylactic vaccines targeting pathogens, mRNA-LNPs have undergone testing in oncology clinical trials for intratumoral expression of combinations of immunostimulatory cytokines or as cancer vaccines [26,27]. Representative examples include mRNA-4157 (V940), an individualized mRNA cancer vaccine encoding 34 specific tumor neoantigens encapsulated by LNPs, are recruiting volunteer patients for clinic trials in melanoma (NCT05933577, Phase 3), renal cell carcinoma (NCT06307431, Phase 2), non-small cell lung cancer (NCT06077760, Phase 3), advanced cutaneous squamous cell carcinoma (NCT06295809, Phase 2/Phase 3) and bladder cancer (NCT06305767, Phase 2).

### 2.3 Lipid nanodiscs

Lipid nanodiscs (LNDs) are disc-shaped nanoparticles featuring a bilayer phospholipid membrane structure, consisting of phospholipids and scaffolding materials. The phospholipids for the preparation of LNDs include dimyristoyl phosphatidylcholine, 1-palmitoyl-2-oleoyl-sn-glycero-3-phosphocholine, cardiolipin among others. The scaffolding materials primarily consist of PEG-phospholipids, proteins, or other polymers [28,29]. The phospholipids and cholesterol are arranged in the center of the LNDs, forming a bilayer membrane structure, whereas the scaffolding materials are distributed along the periphery, defining the shape of the LNDs. LNDs possess hydrophobic cores similar to micelles but lack hydrophilic chambers, hence LNDs are also known as discoidal micelles or bicelles. The sizes can be adjusted according to the prescription with the planar diameter typically ranging from 10 to 70 nm and the thickness varying from 4 to 6 nm [30].

Compared to spherical particles, LNDs exhibit a unique behavior in blood circulation. LNDs tend to tumble and rotate due to the torque forces, significantly enhancing the chances of proximity to vascular endothelial cells. Consequently, LNDs preferentially distribute near the vascular wall, with larger contact areas enhancing adhesion and penetration for targeted lesion delivery [31]. As emerging carriers, LNDs show promise in basic research such as targeted delivery of antitumor and antibacterial drugs [[32], [33], [34]]. For example, intravenously administered LNDs demonstrated superior tumor penetration efficacy over liposomes, enabling the vast majority of tumor cells to be exposed to the STING agonist, thereby triggering robust T-cell activation and effective rejection of established tumors [35].

### 2.4 Other LBNMs

Solid lipid nanoparticles (SLNs), an alternative to liposomes, are aqueous colloidal dispersions with a high-melting-point lipid solid core stabilized by surfactants, with a particle size of 50–1000 nm. They can encapsulate and stabilize both hydrophilic and hydrophobic drugs in their solid matrix, enabling controlled drug release at target sites via crystalline structure maturation [36]. With advantages like low-cost excipients, simple preparation, organic solvent-free, and high drug loading, SLNs are widely used in biomedicine [37].

However, SLNs have certain limitations such as gelatinization at high drug loading, high water diffusion, and drug leakage during storage due to recrystallization. Nanostructured lipid carriers (NLCs) are the advanced generation to minimize some restrictions of SLNs [38,39]. Featuring a solid-liquid lipid core, NLCs use liquid lipids to inhibit lipid crystallization and enhance matrix structural adaptability, thus achieving higher drug loading and lower drug leakage risk.

Lipid nanoemulsions (LNEs) are spherical biphasic liquid droplets (50–500 nm), consisting of an internal dispersed oil phase and an external continuous phase. They form oil-in-water (o/w) or water-in-oil (w/o) emulsions to encapsulate hydrophobic (o/w) or hydrophilic (w/o) active compounds respectively. Emulsifiers (e.g., surfactants) are pivotal for small droplets, as they reduce oil-water interfacial tension. With cost-effectiveness, improved drug bioavailability, robust physical stability, and non-toxicity, LNEs are attractive drug delivery systems [40]. However, their thermodynamic instability, surfactant dependence, and sensitivity to pH/temperature hinder clinical use, making LNEs less favorable than other LBNMs [41].

## 3 Escalating challenges for efficient *in vivo* delivery of LBNMs

In recent years, clinically approved LBNMs, such as Onivyde® (liposomal irinotecan) and Vyxeos® (liposomal daunorubicin and cytarabine), primarily rely on the carrier effect to enhance therapeutic efficacy while minimizing toxic and side effects. They accomplish this by reducing direct contact between the drugs and normal tissues or cells, ensuring the delivery of combination drugs in a fixed ratio, and prolonging the drug’s circulation time in the bloodstream. Indeed, LBNMs, by virtue of their unique nanoscale size, nanostructure, and surface properties, may cause significant changes in drug behavior both *in vitro* and *in vivo*, potentially leading to positive clinical outcomes. However, the complexity and potential safety risks associated with nanoscale effects cannot be ignored and may increase accordingly. LBNMs are actually facing new challenges in their mission to pursue “enhanced efficacy and reduced toxicity” in the therapeutic process.

### 3.1 Unsatisfactory clinical efficacy

The clinical benefits of LBNMs have fallen short of design expectations. For instance, the successful launch of Doxil®, a PEGylated liposomal doxorubicin, is attributed to their ability to effectively reduce the cardiotoxicity of doxorubicin. The PEG modification of the liposome surface prolongs the drug’s blood circulation time, theoretically enhancing its accumulation in tumor sites through passive targeting and thus improving therapeutic efficacy. However, meta-analysis results based on clinical study data indicate that Doxil® have not significantly improved anti-tumor therapeutic effects compared to free doxorubicin, contradicting numerous preclinical study findings [7].

One possible reason for this discrepancy is the uncertainty surrounding the enhanced permeability and retention (EPR) effect in clinical cancer patients. The EPR effect is likely the most pivotal concept in anti-cancer drug delivery systems. It involves hyperpermeable tumor blood vessels allowing large particles to enter and accumulate, evading renal clearance due to their size and remaining due to impaired lymphatic drainage [42]. However, EPR demonstrates substantial variability in humans, differing significantly from murine tumor models in aspects such as growth rate, size relative to the host, metabolic rates, and tumor microenvironment [43,44]. Regarding the two prominent nanomedicines on the market, Doxil® and Abraxane®, they primarily focus on reducing the toxicity of chemotherapeutic agents rather than enhancing their efficacy. Hence, the fundamental rationale behind the design and development of anti-tumor nanomedicines is encountering challenges. Recently, several researchers have challenged that inter-endothelial gap does not account for the accumulation of nanoparticles into solid tumors. Alternatively, they propose that active trans-endothelial pathways are the dominant mechanism for nanoparticle extravasation into tumors [45,46]. Despite the distinct physicochemical properties, such as rigidity and composition, exhibited by gold nanoparticles and LBNMs, the results achieved will bridge the existing knowledge gap in the penetration of nanoparticles into tumors, and facilitate the develop strategies to address the challenge of low clinical translation rate of cancer nanomedicines.

### 3.2 Increasingly prominent side effects

The side effects of LBNMs are also growing concerns. For example, although Doxil® effectively reduce myocardial toxicity, about 50 % patients received Doxil® (50 mg/m^2^ every 4 weeks) experienced palmar-plantar erythrodysesthesia (or hand-foot syndrome), and about 20 % experienced grade 3 palmar-plantar erythrodysesthesia [[47], [48], [49]]. The mechanisms underlying these emerging toxic and side effects are not yet fully elucidated, and the clinical arena still lacks safe and effective management strategies. Unlike small-molecule drugs, LBNMs are more easily recognized by the immune system, leading to adverse reactions such as complement activation, immune cell interactions, and accumulation in non-targeted sites [50,51]. In the early stages of research, LBNMs encapsulated chemotherapy drugs (such as doxorubicin and irinotecan), which suppressed immune function and masked the immunogenicity of the LBNMs [52]. However, when they began to encapsulate biomolecular drugs, their immunogenicity began to emerge, as evidenced by the allergic reactions caused by Pfizer’s BNT162B2 vaccine, which may have been caused by immunoglobulins adsorbed on the surface [53,54]. Furthermore, the bioactivity of carrier materials incorporated within LBNMs cannot be overlooked. PEG, a substance generally regarded as safe and frequently employed to modify the surface of LBNMs to enhance their *in vivo* efficacy, has been documented to elicit an immune response in both animals and humans [[55], [56], [57]]. The widespread occurrence of pre-existing anti-PEG antibodies in the population presents further hurdles for the extensive application of LBNMs [58,59].

### 3.3 Knowledge gaps in intricate *in vivo* delivery processes

**Fig. 2:**
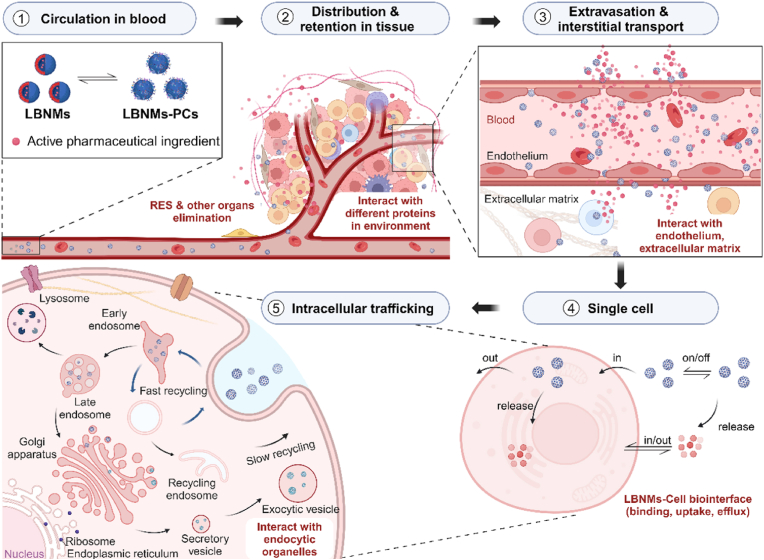
Spatiotemporal transport dynamics of LBNMs from injection to cellular targets. After intravenous administration, LBNMs rapidly disperse within the bloodstream, initiating a cascade of biological processes: 1) Formation of LBNMs-PCs through plasma proteins adsorption; 2) Biodistribution via systemic circulation, with significant accumulation in reticuloendothelial system (RES)-rich organs; 3) Transvascular transport into tissue interstitium through paracellular/transcellular diffusion or convective flow, followed by interactions with extracellular matrix components; 4) Interstitial migration via diffusion/convection to achieve cellular proximity, involving receptor-mediated binding, endocytic uptake, and potential efflux mechanisms; 5) Intracellular trafficking through endosomal/lysosomal compartments with subsequent cargo release or degradation. Created with BioRender.com.

Most importantly, the complex *in vivo* processes of LBNMs encompass a delivery mechanism that remains enigmatic and largely unexplored, constituting a significant knowledge gap in this field. Currently, there are well-established research methodologies for exploring the *in vivo* behavior of small molecular drugs or conventional injections [60]. However, akin to other nanomedicines, the *in vivo* delivery of LBNMs presents an unusually complex scenario. The journey *in vivo* involves multiply process such as blood circulation, organs distribution, tissues penetration, cellular recognition and intracellular trafficking [61] (Fig. 2). As nanosized exogenous substance, LBNMs engage in intricate interactions with the immune system upon entering the body, further convoluting the *in vivo* delivery process and potentially altering their safety and efficacy [62]. Furthermore, LBNMs inevitably undergo bio-nano interface interactions with various biological milieu (blood, lymph, interstitial fluid and cellular components, *etc*.), significantly altering their surface properties [63]. These alterations cause a divergence in pharmacokinetics, immunogenicity, efficacy, and safety profiles from what is anticipated based on *in vitro* studies. Therefore, efficient *in vivo* delivery of LBNMs is facing new challenges, necessitating a nuanced and rational understanding of their fate *in vivo*.

### 3.4 The critical unmet need for robust IVIVC models in LBNMs

Establishing robust IVIVC for LBNMs is pivotal to deciphering their *in vivo* fate and enabling precise predictions through *in vitro* models. Such advancements would not only address the translational challenges hindering LBNMs but also inspire innovative solutions to optimize their therapeutic efficacy. By bridging this critical gap, researchers can systematically link LBNMs design parameters-such as surface chemistry, size, and charge—to their dynamic interactions with biological systems, ultimately guiding the rational development of next-generation formulations that overcome biological barriers.

Traditionally, IVIVC has served as a cornerstone for predicting drug behavior by correlating physicochemical properties with plasma concentration profiles via dissolution testing. For LBNMs, however, this paradigm faces fundamental limitations: conventional IVIVC methods, which prioritize dissolution kinetics, fail to account for dynamic bio-nano interactions such as protein recognition, cellular uptake, and immune interplay. This limitation is particularly pronounced in injectable LBNMs, where intravenous administration triggers immediate interactions with blood components, reshaping LBNMs’ biological identity in ways that *in vitro* dissolution assays cannot replicate. Additionally, LBNMs designed for controlled release—such as nucleic acid-encapsulating systems—exhibit release profiles divergent from small-molecule drugs, rendering standard dissolution models inadequate. The intrinsic bioactivity of LBNMs, encompassing surface modifications, lipid composition, and targeted ligand interactions, further exacerbates this complexity.

While IVIVC modeling for small-molecule drugs has reached relative maturity, research in this domain for LBNMs remains notably lagged. This disparity highlights the pressing need for tailored IVIVC frameworks that account for LBNMs’ unique physicochemical and biological complexities. To address this gap, the field is increasingly shifting toward integrated multi-disciplinary frameworks that synergize physiologically based biopharmaceutics modeling, artificial intelligence-driven predictive algorithms, and real-time characterization of dynamic nanocarrier-biology interactions. By embracing this cross-disciplinary approach, researchers can bridge the long-standing gap between *in vitro* predictions and *in vivo* outcomes, thereby accelerating the clinical translation of LBNMs and unlocking their full therapeutic potential.

## 4 A quick guide for IVIVC

IVIVC modeling is one of the most classic and useful model-informed drug development (MIDD) approaches, a powerful tool to support drug development and regulatory review [64]. The earliest application of MIDD approaches can be traced to the 1990s, which included the application of methods such as IVIVC model, pharmacokinetic/pharmacodynamic (PK/PD) model, and population pharmacokinetics (popPK) model. Since the 21st century, MIDD has undergone exponential growth, expanding its applications to inform dissolution specifications for biowaivers, optimize dose selection/clinical trial design, characterize safety/efficacy profiles, and guide regulatory/policy decisions. Models such as physiologically based pharmacokinetic modeling (PBPK), physiologically based biopharmaceutics modeling (PBBM), exposure-response modeling, quantitative systems pharmacology modeling (QSP), real time release test (RTRT), and artificial intelligence (AI)/machine learning (ML) modeling are applied to facilitate drug development. In recent years, physiologically-based nanocarrier biopharmaceutics (PBNB) model is developed for reverse-engineering the *in vivo* drug release of LBNMs [[65], [66], [67]]. Depending on the needs, a single modeling approach or a combined modeling approach such as PBPK-based IVIVC, PBNB-based IVIVC can be used to enhance drug development.

IVIVC allows assessment of *in vivo* performance changes resulting from pre- and post-approval changes in formulation attributes, manufacturing processes, equipment and sites based on *in vitro* drug release data. It can also serve as a surrogate for bioequivalence studies [68,69]. FDA guidance of extended-release oral delivery systems defines IVIVC as “a predictive mathematical model describing the relationship between an *in vitro* property of a dosage form and a relevant *in vivo* response” [68]. The *in vitro* property and the relevant *in vivo* response typically refer to the *in vitro* drug release rates, and the *in vivo* drug absorption/drug release rates, respectively. The *in vivo* drug absorption/drug release rates are typically estimated based on the PK profiles using a deconvolution technique, or a PK parameter such as the maximum plasma drug concentration (C_max_) or the cumulative area under the curve (AUC). This definition emphasizes a “predictive” aspect of the relationship which allows the use of an *in vitro* drug release to predict the corresponding *in vivo* drug release. In contrast, the United States Pharmacopeia (USP) defines IVIVC in a broader way: “the establishment of a rational relationship between a biological property, or a parameter derived from a biological property produced by a dosage form, and a physicochemical property or characteristic of the same dosage form”. The *in vivo* response in this definition is “any biological property produced by a dosage form” which is not limited to the traditional PK profile and parameters. Accordingly, semi-quantitative or rank-order relationships between *in vitro* and *in vivo* is covered (*in vitro-in vivo* relationship, IVIVR).

**Fig. 3:**
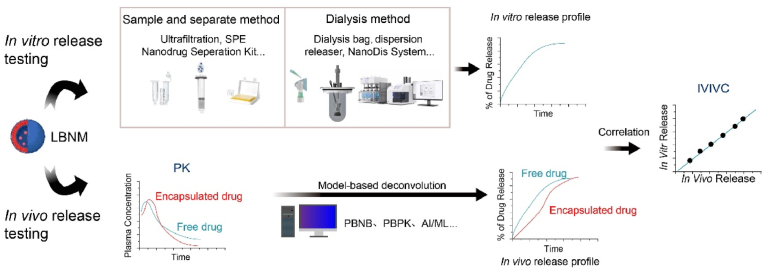
IVIVC development for LBNMs.

Establishing IVIVCs requires a biorelevant *in vitro* assay reflecting the release mechanism of the carrier and an accurate analysis of the clinical PK data. Accordingly, a reliable dissolution method is needed. Also, methods with good accuracy are needed to generate *in vivo* PK data of both the encapsulated and the non-encapsulated fraction of the drug. Furthermore, the PK data has to be appropriately deconvoluted before model building. Fig. 3 illustrates the general workflow for establishing IVIVC for LBNMs, encompassing *in vitro* dissolution, PK sample separation, and model-based deconvolution.

There are five levels of IVIVCs describing *in vitro-in vivo* drug release correlations: Level A, Level B, Level C, Multiple Level C, and Level D. Level A IVIVC is a point-to-point correlation between *in vitro* release and *in vivo* absorption. It is the only IVIVC which is helpful for regulatory compliance. Level A IVIVC is commonly established by developing formulations with three varying release rates (fast, medium, and slow) and obtaining *in vitro* release and *in vivo* absorption profiles through deconvolution for the respective formulations. Level B correlation compares a mean *in vitro* drug release time to a mean *in vivo* drug absorption time. Level C correlation is a single point relationship between one drug release time point (e.g., time for 50 % release) and one PK parameter (AUC, C_max_, or T_max_) which is normally only used during preliminary formulation development. Level D correlation is not a formal correlation since it is only a rank order or a qualitative comparison.

For oral extended release formulations or parenteral long-acting injectables such as microspheres, *in situ* forming implants, the *in vivo* deconvolution required for Level A IVIVC development can be achieved using an *in silico* model-based method, such as Wagner-Nelson and Loo-Riegelmann, or using a non-model-based general numerical method [10]. However, these methods may not apply for LBNMs given their complex *in vivo* process. Plasma PK profiles of LBNMs is widely dominated by the drug release, kinetics of the drug-protein transfer [70], endothelial adsorption, particles adhering to blood cells [71] and the tissue accumulation of the carrier which includes extravasation, or disruption/elimination of the carrier [72,73]. The endothelial adsorption potentially causes significant delay between the end of the infusion and T_max_ following the infusion of LBNMs [67,74]. Moreover, the drug release of LBNMs is complicated which is associated with complicated release mechanisms including dispersion, dissolution, dissociation [75,76], and transfer of small-molecular drugs from the carrier to proteins [70] and membranes [77]. Accordingly, the deconvolution of LBNMs PK data is challenging. The success rate for drug release-based LBNMs IVIVC development is consequently low. Moreover, conventional IVIVCs typically need a lot of resources to validate and implement, which further hampers the IVIVC development for LBNMs. Therefore, advanced complex *in silico* models with more flexibility and utility, such as PBPK modeling, PBNB modeling, and AI/ML, *etc*. are needed to facilitate the establishment of IVIVCs for LBNMs. These models leverage published data, take physicochemical, pharmacological/biopharmaceutical, and physiological factors affecting both dissolution as well as absorption of drugs into consideration, serving as powerful tools to advance IVIVC [14]. In addition, since not all the LBNMs are designed to release drugs in a traditional way, the IVIVC development for LBNMs cannot be limited to the conventional correlation between *in vitro* and the *in vivo* drug release, but also the correlation between formulation characters such as particle size, cellular uptake *in vitro*, *etc*. and their *in vivo* performances such as RNA silencing effect, *etc.*, depending on their purpose of use, *i.e.* IVIVR.

## 5 The evolving paradigm of IVIVC for LBNMs

### 5.1 In vitro dissolution testing and modeling

A desirable *in vitro* release method should reflect drug release mechanism, have short assay times, and have capability to predict *in vivo* performance and discriminate different formulations. Since the drug release of LBNMs strongly depends on the microenvironment, the *in vitro* testing conditions such as composition of release medium, shear forces, *etc*. should reflect the *in vivo* situation [78,79]. In addition, unlike traditional dosage forms which typically require sink-conditions, non-sink conditions sometimes are used for nanomedicines applied via the subcutaneous or intramuscular route where the drug encapsulated slowly releases in a limited volume of physiological fluid [80,81]. Currently, there have been several methods reported for the *in vitro* drug release testing of LBNMs [75,82], including sample and separate method, dialysis-based methods and *in situ* detection methods/RTRT.

#### 5.1.1 Sample and separate methods

Commonly used separation techniques include filtration [83], centrifugal ultrafiltration [84], ultrafiltration [85], preparative ultracentrifugation [86], solid phase extraction (SPE) [87], and asymmetrical flow field flow fractionation [88]. In recent years, a novel PEG-scFv-based separation method which is currently commercially available as Nanodrug Separation Kit (Manufactured by Andisso) has been developed for PEGylated liposomes. This technology adopts anti-PEG single chain variable fragment antibody (PEG-scFv) to sediment PEGylated liposomes. Only simple incubation and low speed centrifugation are needed to achieve accurate and efficient separation of released drugs and liposomal drugs in medium. This method is suitable for both *in vitro* quality assessment, drug release testing and *in vivo* pharmacokinetics evaluation of PEGylated liposomes and possible other PEGylated nanocarriers [89]. Accordingly, the PEG-scFv-based separation method can serve as an alternative approach of traditional methods in the *in vitro* release testing of such formulations.

NanoDis system is another promising technology for samples and separations. It is an automated dissolution apparatuses developed by Agilent for nanoparticle dissolution testing. It can adequately and repeatably separate the carriers from the dissolved drug prior to sample analysis through a hollow fiber filter [90]. Research into the use of NanoDis System highlights advantages over traditional release methods, such as reduced manual error and significant time savings. NanoDis therefore may be widely used in the *in vitro* release testing of LBNMs.

#### 5.1.2 Dispersion releaser technology

The dispersion releaser technology is widely used and have shown desirable ability to establish IVIVCs for nanomedicines [75]. This method was developed in 2013 at Goethe University and became commercially available in the beginning of 2018 (manufactured by Pharma Test Apparatebau AG) [82]. It comprises a sample holder cell for dispersed dosage forms (donor compartment), from where the free drug substance diffuses through a membrane in the acceptor compartment. Sample collection from both the accepter compartment and the donor compartment is allowed. The donor chamber is agitated by a paddle stirrer which applies controllable shear force to the nanoformulation. The propulsion is transmitted to a magnetic stirring device in the acceptor compartment. This set-up facilitates a more efficient membrane transport of the drugs compared to other dialysis-based setups [75]. The selection of material of the membrane molecular weight cut off is critical for the drug release. However, no matter what kind of membrane is used, membrane transport normally causes a delay in drug release since this process is typically rate limiting [75]. According, the release profiles should be normalized by the rate constants of membrane diffusion [91]. When using biopredictive release medium where drug degradation occurs during release testing, the drug release profile should also be corrected for drug degradation through *in silico* models. In addition, dispersion releaser along with biopredictive and/or non-biopredictive release media is demonstrated to be a time-resolved and sensitive technology for studying drug release mechanisms of liposomes [92]. Through this method, the release mechanism of liposomes TLD-1, Lipodox®, and Caelyx® was found to be driven by the dissolution of doxorubicin nanocrystals within the liposomal core [92]. The dispersion releaser technology in combination with a four-step model also enabled the measurement of the transfer of drugs from liposomes to proteins like PC [70]. Accordingly, dispersion releaser serves as a desirable technology for IVIVC development for LBNMs (details about IVIVC development refer to Section 5.4).

#### 5.1.3 In situ detection methods/RTRT

*In situ* detection methods/RTRT are live measurements which continuously monitor the drug release in the release medium without causing drug or formulation loss. This method is particularly suited for rapid releasing LBNMs. Measurements can be realized with the aid of analytical detection methods such as UV/Vis-detection, fluorescence, IR/Raman, potentiometry and calorimetry. Such methods do not require sampling, separation or liquid handling, allows the use of small volumes of medium, and can minimize human error [91]. For this aspect, *in situ* detection methods are superior to the traditional sample and separate methods and the dialysis-based methods. However, such methods have the drawbacks of the quantification limits which are primarily determined by analytical properties of the drug substances, and complex validation procedures. Besides, the methods are cost-efficient only when the release testing is used for quality control of continuous manufacturing approaches.

To establish a IVIVC with more desirable predictability and to better understand the drug release mechanisms, the *in vitro* drug release data obtained using the abovementioned methods typically need to be processed via model fitting based on drug release kinetics models or mechanistic models prior to IVIVC development [93]. The commonly used release kinetics models include zero-order kinetics model, first-order kinetics model, Weibull release kinetic model, Double Weibull release kinetic model, Higuchi model, Hixson-Crowell, Hill, Makoid-Banakar, and Korsmeyer-Peppas model, *etc*. Mechanistic models include Toroidal, All-or-None, and Biomembrane models *etc*. Detailed information about the traditional models can refer to a previous review paper [93]. Given the unique complex drug release profiles of LBNMs, some customized models such as three-parametric reciprocal powered time (3RPT) model have to be used [94]. As mentioned above, for drugs tested using dialysis methods and experiencing degradation following drug release, the release profiles should be corrected for permeation and degradation using appropriate models. Other corrections should be made as well if necessary [94].

### 5.2 Separation method for PK samples

With regards to the *in vivo* data used for development of an LBNMs IVIVC/IVIVR, recent US-FDA guidelines require a quantification of the encapsulated and non-encapsulated fraction of the drug from the blood plasma [95]. This demands a sensitive and accurate bioanalytical separation method which can separate free drug and/or nanocarriers from the plasma. Most of the commonly used methods overlap with the methods used for the *in vitro* release testing [69], including ultrafiltration, solid-phase extraction, size-exclusion chromatography, PEG-scFv-based separation method, *etc* [89,[96], [97], [98], [99]]. However, none of the current methods are perfect since they are either being cumbersome, or having carrier adhesion issue, or potential drug leakage. All these affect the accuracy of the *in vivo* PK data. PEG-scFv-based sample and separation method was shown to generate more accurate *in vivo* PK data with lower error bars compared traditional methods. It is therefore a desirable method for PEGylated LBNMs. Such kind of novel facile and accurate separation methods are needed for other LBNMs.

### 5.3 Model-based deconvolution and correlation

As mentioned in Section 4, conventional deconvolution methods are not applicable for the PK data of LBNMs. A few advanced computational models have been used as alternative strategies for deconvolution*, in vitro* data simulation and implementation, and IVIVC development (i.e. PBNB model, PBPK model and AI/ML models). Such models are time- and cost-saving since they can be built based on published data. The models take both formulation and biological factors into consideration, accordingly, revolutionize the understanding and prediction of LBNM ADME parameters (absorption, distribution, metabolism, and elimination), PK, release mechanisms, efficacy, toxicity.

#### 5.3.1 PBNB modeling

Most of the current successful IVIVCs of LBNMs are established based on PBNB model. The PBNB model was first developed in 2020 [67]. It is used to deconvolute the pharmacokinetic profiles of nanodrugs to achieve release profiles of free and carrier-bounded fraction of the drug, offering a standardized strategy for determining the *in vivo* release of LBNM. PBNB models are typically established based on the existing clinical data of various nanomedicine. The PK data is extracted, analyzed and then used for the model development using a series of software. PBNB models takes various *in vivo* factors into consideration, including patient population [100], unique PK characters of LBNMs such as prolonged vascular transit period of liposomal formulations observed only in patients, *etc* [66,67]. In addition, the PBNB model effectively separates formulation-related effects from drug-related parameters.

#### 5.3.2 PBPK modeling

PBPK modeling is a powerful tool in the simulation of ADME characteristics of the LBNMs. It has the ability to characterize and predict the PK, toxicity, efficacy, and target exposure of various types of LBNMs [101]. The formation of a PBPK model requires species-specific, patient-specific and compound/formulation-specific data/parameters, and partition coefficients in different tissues. The PBPK modeling can be used to assess the clinical relevancy of *in vitro* characterizations, determine bioequivalent specifications for *in vitro* testing parameters, identify critical quality attribute, develop bio-predictive dissolution method and further establish IVIVC. Once established, PBPK-based IVIVC can virtually simulate local and systemic drug exposure based directly on formulation parameters. When establishing PBPK models for the LBNMs, LBNM-specific parameters such as the Hill coefficient, phagocytic cell uptake rate (typically following Hill function), drug release rates, *etc*. should be included. Moreover, since the ADME processes of LBNMs have unique complexity, some of the commonly used PBPK modeling software packages such as ADAPT, Stella, SAAM II, Simcyp and GastroPlus do not provide sufficient flexibility and capability to deal with LBNMs. Powerful tools such as Matlab-Simulink and ACSL/acslXtreme may be preferable [102].

#### 5.3.3 AI/ML modeling

AI technologies simulate human intelligence to conduct complex tasks via learning, reasoning, and problem-solving [103,104]. ML develops programs for modeling and predicting, *etc*., based on historical data and experiences. AI/ML modeling leverages extensive published data to build robust predictive models, allowing the exploration of unknown relationship between *in vivo* process and physiochemical properties of various drug formulations. Such models offer the most powerful data-driven strategies in accelerating and revolutionizing the nano-drug development. One recent study investigated the potential of utilizing multiple AI/ML models (i.e. Logistic Regression, Support Vector Regression, Random Forest, XGBoost, LightGBM, and Deep Neural Networks) for predicting tissue distribution and tumor delivery of nanoparticles. Physicochemical properties of nanoparticles cancer treatment strategies and dosing regimens with the dataset from Nano-Tumor Database were used as inputs in the predictions. Deep Neural Networks were shown to have superior predictive capability. Furthermore, the researchers converted the Deep Neural Network-based model into a user-friendly web dashboard (Nano-AI-QSAR data analysis), potentially serving as a useful high-throughput pre-screening tool for efficient and rational nanoparticle development without the use of animals [105]. There also have been studies incorporating AI/ML models to enhance predictive capabilities of traditional MIIDs for LBNMs [106,107]. In a wide sense of the word, PBPK and PBNB are ML tools [108].

### 5.4 IVIVC/IVIVR cases of LBNMs

Despite all the above-mentioned advances in *in silico* modelling and *in vitro* release methods only countable successful IVIVCs/IVIVRs are developed for LBNMs. The cases are discussed below.

#### 5.4.1 PBNB-based IVIVC

In a recent study, researchers established robust patient-specific IVIVCs with desirable predictive power based on Caelyx®, Lipodox® and a liposomal doxorubicin formulation (TLD-1) [92] The three formulations had different liposome membrane integrity, core crystallinity, particle size and surface charge. *In vitro* drug release data was obtained using the Pharma Test dispersion releaser technology in combination with a PTWS 120D dissolution tester following the description in the USP (2016). The release medium was PBS buffer containing 10 % FBS. The obtained *in vitro* release profiles were normalized through modeling doxorubicin *in vitro* degradation and membrane permeation to eliminate the analytical error caused by the dialysis membrane and drug degradation. Average *in vivo* release of TLD-1 was obtained through deconvolution of individual clinical pharmacokinetic data using the PBNB model designed with Monolix2020R1. The IVIVC was established by correlating the *in vivo* and *in vitro* free doxorubicin released at specific time points through stretching and shifting methods. Despite a narrow predictive window of the developed IVIVC model due to the similar release profiles of TLD-1, Lipodox®, and Caelyx®, *in vitro* release method can effectively discriminate formulation performance changes and predict clinical AUC rank order based on the *in vitro* rank order of release. The presence of serum (FBS) in release medium was found to enhance the predictive accuracy of IVIVC models. The coefficient of the IVIVCs obtained with 10 % FBS was excellent (R^2^ > 0.97) while the R^2^ of IVIVCs obtained with PBS was only 0.71–0.94. In addition, the dynamic high-shear environment provided by *in vitro* dispersion releaser was believed to affect the formation of PC on liposomal surface [109] and consequently played an essential role in the successful IVIVC development [92]. This study establishes the critical role of PC in LBNM IVIVC development.

Modh et al. also successfully established a Level A IVIVC based on the dispersion release technology (*in vitro* release under biorelevant conditions) along with a hybrid custom-made PBNB model (PK data were from first-in-men clinical trials). Four different types of injectable doxorubicin LBNM formulations, *i.e.* doxorubicin PLGA nanoparticulate formulations, NanoCore-7.4 and NanoCore-6.4 prepared using double emulsion solvent evaporation method were used to develop the IVIVC model. The IVIVC model were then validated using NanoCore-7.4 and drug product Lipodox. *In vitro* and *in vivo* release of Lipodox and NanoCore-7.4 demonstrated a good correlation [100].

Another group developed a rank order IVIVR based on two doxorubicin drug formulations with different quality and material attributes and resultantly distinct release and accumulation behaviors using the same strategy [109]. The *in vitro* data were obtained using dispersion releaser and were corrected by the error caused by membrane permeation and degradation through model-based normalization. The *in vivo* deconvolution of the release and unreleased drug PK was performed through PBNB model based on literature data of the two formulations. The input parameters included the elimination and distribution parameters of the free drug, and carrier-associated drug, *etc.* A liver compartment was introduced to the model to predict liver exposure of the formulations based on the carrier-associated and free drug fractions [65]. The developed model allows an acute estimation of the drug concentrations (including released and encapsulated fractions) in plasma, liver as well as other organs. The drug release mechanism *in vivo*, and the impact of quality attributes of formulations on bioavailability and toxicologically relevant exposure levels were determined, which allows the establishment of a design space for future LBNM development and an early screening of formulation modifications.

#### 5.4.2 PBPK-based IVIVC

Attempt was made by a group to investigate IVIVC of the distribution and PK of glycol-coated gold nanoparticles (PEG-AuNPs) based on PBPK modeling [110]. Human cell lines TH1, A549, Hep G2, and 16HBE were employed for *in vitro* internalization and exclusion investigation. An equal volume of 100 % FBS was used to dilute nanoparticle stock solution to allow the formation of bio-relevant PCs. Rats were used to study the biodistribution and PK. *In vitro* PK of PEG-AuNPs were modeled as first-order rate processes since tissues and organs are usually assumed to be first-order reaction kinetics. The resulting parameters were then translated into the PBPK-related parameters using a perfusion rate-limited PBPK model through SimBiology toolbox and were used to predict the particle biodistribution *in vivo*. It was found that the cell lines used cannot simulate the active uptake of LBNMs by phagocytes *in vivo* due to the absence of natural barriers. It is therefore suggested to use membrane-limited PBPK models for LBNMs [111]. Such models consider the phagocytic cell-mediated active internalization as the dominating route of nanoparticle uptake into tissues, and accordingly, the total particle burden can be correlated with the phagocyte number in the corresponding tissue.

#### 5.4.3 IVIVC development-based high-throughput technologies

IVIVC development for siRNA delivering nanomedicines is particularly difficult compared with other LBNMs due to the cost associated with generating large in vitro-in vivo data sets. To address this issue, high-throughput techniques was utilized in one report. A direct IVIVC between *in vitro* transfection results and in animal gene silencing mediated by siRNA was developed. The high-throughput technique used allowed the examination of hundreds of siRNA LBNMs for their transfection efficiency based on multiple experimental systems. The type of cell used for *in vitro* transfection study was found to affect correlations, with primary hepatocytes and HeLa cells the most suitable cell lines for IVIVC development for siRNA nanomedicines. For formulation property side, the *in vivo* silencing potential can only be predicted partially by siRNA entrapment efficiency. The zeta-potential and particle size (<300 nm) of siRNA nanoformulations surprisingly did not show any correlations with *in vivo* silencing [112].

In conclusion, PBNB model and the dispersion releaser appear to be relatively more promising strategies allowing drug-dependent IVIVC development for LBNMs. However, as mentioned above, this only resulted in five successful cases of LBNM IVIVC, which is a very small number for a wide-using dosage form. Moreover, most of the IVIVC models have prediction limitations, such as the limitation in predicting carrier half-life, *etc* [100]. This may, to certain extent, be due to the inadequate understanding of PCs, and/or other biological coronas. The importance of PCs is addressed in two IVIVC cases [92,110] where *in vitro* conditions allowing the forming of bio-relevant PCs on the surface of LBNMs are used to minimize the effect of PCs in order to develop desirable IVIVCs [110]. This indicates that PCs may be one of the most critical factors in LBNM IVIVC development. Unfortunately, the two studies did not deeply study the effect of PC on LBNM performance. The following section gives a comprehensive review of PCs (including the formation, composition and evolution of PC, biological implications, and methodologies of preparation, separation, identification as well as characterization), which will be helpful to better understand the role of PCs in bridging the gap between *in vitro* characterization and *in vivo* behavior, and may inspire a useful methodology of studying the roles of PCs in IVIVC development.

## 6 Protein coronas

**Fig. 4:**
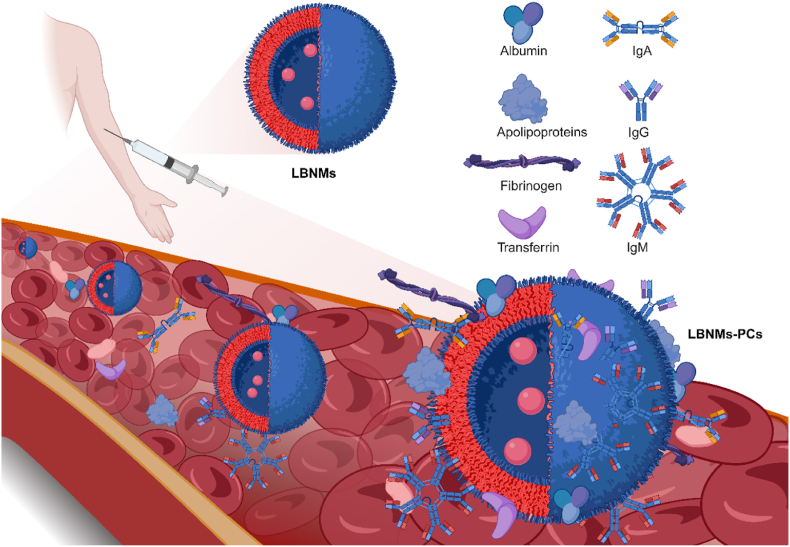
Formation of PCs on LBNMs during systemic circulation. After intravenous injection, LBNMs immediately adsorb plasma proteins (e.g., IgG, IgM, albumin, transferrin, apolipoproteins, fibrinogen) to form dynamic PCs. Created with BioRender.com.

Although the synthetic LBNMs process a well-defined size and surface composition, these characteristics might undergo transformation immediately upon entering biological fluids. The surface of LBNMs transiently adsorbs a dynamic layer of proteins, collectively known as PCs [113] (Fig. 4). The original chemical or biological functions conferred to the LBNMs are immediately masked or superseded by PCs, thus conferring new biological properties [114,115]. PCs has been shown to determine the biological identity and *in vivo* fate of LBNMs, thus providing another perspective for the IVIVC of LBNMs beyond dissolution.

### 6.1 Formation, composition and evolution of the PCs

The formation of PCs is entropy-driven, spontaneous, and inevitable. Due to their enlarged relative surface area and high surface free energy, LBNMs inherently attract and bind a diverse array of proteins through a multitude of interactions, including hydrogen bonding, electrostatic adsorption, hydrophobic interactions, and Van der Waals forces [116].

The formation of PCs is dynamic. An initial layer of loosely bound, high abundance proteins (soft corona) temporarily cover the surface and then gradually give way to a more stable layer of tightly bound proteins with high binding affinity and low desorption rates (hard corona) [117]. This process is in accordance with the Vroman effect [118]. Similarly, as the proteins-coated engineered LBNMs traverse different biological fluids (e.g. leaking from blood vessels, or being internalized by cells), PCs undergoes dynamic evolution and reequilibration, endowing the LBNMs with new biological properties that has the potential to significantly alter the biological fate and behavior.

Meanwhile, the formation of PCs is controllable. Theoretically, the properties of PCs formed on lipid nanomedicines surfaces, including number, type, thickness, and adsorption saturation time, are governed by various factors like particle size, shape, surface modification, and environmental pH values [119]. Consequently, the *in vivo* generation of PCs can be actively modulated by adjusting these characteristics of lipid nanomedicines surfaces [120].

### 6.2 Preparation, separation, identification and characterization of PCs

#### 6.2.1 Preparation

The *in vivo* acquisition of PCs provides the most accurate results. For instance, a high dose of LBNMs can be directly injected into the tail vein of the experimental animal, the PCs can be obtained after appropriate separation of the serum samples. Alternatively, to accurately mimic *in vivo* microenvironments, LBNMs of constant concentration are incubated within a tailored environmental or biological matrix (encompassing plasma, mucus, cell lysate or conditioned medium) for a predetermined duration *in vitro*. During the period, PCs naturally form on the surface of LBNMs. For a comprehensive understanding of the PCs formation process, complex whole blood or cell culture medium are often employed as the matrix. Alternatively, when the focus is on elucidating the specific interactions between LBNMs and a specific blood protein, a conditioned medium containing a single protein (e.g., albumin, apolipoprotein, transferrin, or immunoglobulins could serve as the optimal matrix [[121], [122], [123]].

#### 6.2.2 Separation

Effective PCs isolation from unbound biomolecules in complex fluids is vital for accurate protein content analysis of LBNMs corona. Centrifugation (including ultracentrifugation and sucrose cushions) represents a standard method for PCs separation based on the density difference between the samples and the medium [124,125]. However, it is a rugged method and aggregates or supermolecules with high molecular weight are easily be collected during centrifugation. In addition, centrifugation is extremely inefficient for collecting small-sized nanocarriers. Zhang et al. [126] provided a PEG-scFv-based affinity chromatography technique for rapid separation of PCs with high efficiency and quality. PEG-scFv is a His-tagged anti-PEG single-chain variable fragment designed specifically to capture PEGylated-liposomes within biological medium. It sequentially binds the Ni-NTA beads via His tag to eliminate the endogenous contaminants such as unbound free proteins and protein aggregates. This innovative method demonstrates superior collecting efficiency, yields more bioinformation and reduces false risks on the identification and quantification of PCs compared to the traditional centrifugation and size-exclusion chromatography. Table 1 summarizes a comparison of key methods for the separation of PCs.

**Table 1:** Comparison of key techniques for protein coronas separation and characterization.

Specific Method	Purpose	Advantages	Disadvantages
Ultracentrifugation	Separate LBNMs-PCs from free proteins	Widely accessible	Harsh (PCs distortion/aggregation); Low efficiency for small LBNMs; Non-specific co-precipitation
PEG-scFv Affinity Chromatography	Separate LBNMs-PCs from free proteins	High specificity/efficiency; Gentle (preserves PCs); Reduces background	Only for PEGylated LBNMs; Needs scFv/Ni-NTA beads
TEM	Observe LBNMs-PCs morphology	High resolution (nano-subnano scale)	Vacuum + complex sample prep (artifacts); No in-situ/physiological observation
Cryo-EM	Observe LBNMs-PCs under near-physiological conditions	High resolution; Preserves native structure	Extremely high cost; complex operation/data analysis
AFM	Observe LBNMs morphology + measure PC thickness	Operable in atmospheric/liquid (near physiological); 3D topology + PC thickness	Slow scanning; μm-scale range; needs flat sample
NTA	Monitor LBNMs size + measure concentration	Single-particle tracking; Concurrent concentration	Affected by Brownian motion; low sensitivity for low-concentration samples (<10^7^ particles/mL)
DLS	Monitor LBNMs size; estimate PCs thickness/protein quantity	Simple, fast, low sample (μL); in-situ real-time	No monodisperse/polydisperse distinction; Impurity/bubble interference; Only average size
BCA Assay	Determine total PCs mass	Simple, broad protein compatibility	No protein type/proportion distinction; interfered by reducing agents (e.g., DTT)
SDS-PAGE	Qualitative/semi-quantitative PCs composition	Simple, low cost; separates by molecular weight	Semi-quantitative; Needs MS for unknown proteins; Denaturing masks native states
LC-MS/MS	Quantify PCs components + detect post-translational modifications	High separation/sensitivity (ng/mL); Identifies unknown proteins + amino acid sequences	High cost; Low reproducibility (needs standardization)
FTIR	Characterize PCs protein structure	Simple; no labeling; provides functional group + secondary structure information	Limited resolution; no tertiary/quaternary details; water/impurity interference
ITC	Characterize PCs-LBNM binding kinetics/affinity	No labeling; measures full thermodynamic parameters; reveals mechanisms	Large sample (μmol); Long cycle; Low sensitivity for low-affinity (Kd > 10^−6^ M)
SPR	Characterize PCs-LBNM binding kinetics/affinity	Real-time label-free; provides ka/kd/Kd; high resolution	High cost; Molecule immobilization (may affect activity); Refractive index/temperature interference
MD Simulations	Obtain PCs-LBNM interaction dynamics	Simulates molecular processes; predicts mechanisms; complements experiments	Relies on parameters/force fields; high computational cost; needs experimental verification

#### 6.2.3 Identification and characterization

**Fig. 5:**
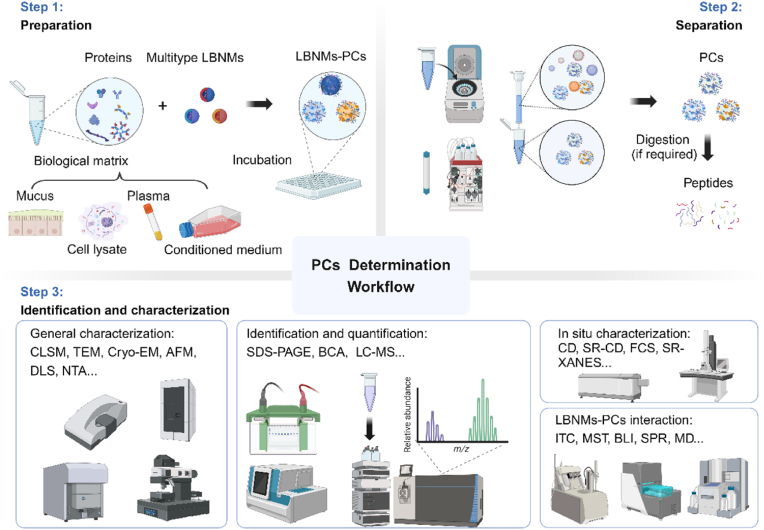
Workflow for PCs characterization on LBNMs. The workflow includes: 1) Sample preparation (incubation of LBNMs with biological matrices). 2) LBNMs-PCs separation. 3) Identification and characterization (e.g., composition, *in situ* properties, interactions). Created with BioRender.com.

Once isolated, the alteration of LBNMs by PCs in basic physicochemical properties (morphology, size and surface charge) can be identified and characterized with diverse techniques [127] (Fig. 5). For example, the morphological change of LBNMs can be direct visualized using confocal laser scanning microscopy (CLSM), transmission electron microscopy (TEM) and Cryo-EM [128,129]. Atomic force microscopy (AFM) serves as a potent tool to observe the morphologies and thickness of PCs coated on LBNMs [130]. Nanoparticle tracking analysis (NTA) or dynamic light scattering (DLS) are useful techniques to monitor the change of hydrodynamic size. Additionally, DLS can provide information on the thickness and number of proteins present on the surface of LBNMs [130,131]. The bicinchoninic acid (BCA) assay can be employed to determine the total mass of the PCs.

A more precise quantitative measurements on the composition of the PCs can be identified with sodium dodecyl sulphate polyacrylamide gel electrophoresis (SDS-PAGE), capillary electrophoresis–mass spectrometry (CE-MS) and liquid chromatography–mass spectrometry (LC-MS) [132,133]. Fourier-transform infrared (FTIR) spectroscopy analysis can be used to characterize the structure of the bound proteins. In situ techniques such as circular dichroism (CD), fluorescence correlation spectroscopy (FCS), synchrotron radiation-based CD (SR-CD) and synchrotron radiation-based X-ray absorption near edge structure (SR-XANES) provide structural information on the orientation of the biomolecules in PCs [134]. Isothermal titration calorimetry (ITC), microscale thermophoresis (MST), biolayer interferometry (BLI) and surface plasmon resonance (SPR) [135] can characterize the kinetics parameters and binding affinity between PCs and LBNMs. Critical information regarding the interaction dynamics between LBNMs and the PCs can be obtained with molecular dynamics (MD) simulations [136].

### 6.3 Some key components in protein coronas and their general biological implication

Numerous plasma proteins have been reported to bind to LBNMs, some crucially modulating pharmacological, toxicological and therapeutic profiles, while others exhibiting minimal effects. Functionally, the composition of PCs can be broadly divided into opsonins and de-opsonins. Opsonins, including complement proteins, immunoglobulins, fibrinogen, and fibronectin, facilitate phagocytosis by immune cells, leading to rapid clearance of LBNMs from the bloodstream and compromising drug delivery efficacy [137]. In contrast, de-opsonins, represented by albumin, weaken the interaction with cell membranes and protect against phagocyte recognition, thereby prolonging blood circulation and enhancing drug delivery of LBNMs [138]. Kostarelos et al. [139] investigated and characterized the *in vivo* PCs formation of Caelyx (PEGylated liposomes encapsulating doxorubicin) in ovarian carcinoma patients. Their findings revealed that immunoglobulins constituted the most prevalent class of proteins, comprising 28 % of the total proteins content, followed by lipoproteins at 9 % and complement proteins at 4 %, respectively, in agreement with their prior studies in mice [140]. Zhan et al. [126] deciphered PCs composition of PEGylated liposomes using scFv-based affinity chromatography and similarly found apolipoproteins, immunoglobulins and complements to be enriched within the PCs. Pattipeilihu et al. [141] identified the enrichment and high abundance of apolipoproteins on the surface of anionic AmBisome liposomes using a photoaffinity-based approach. Remarkably, although these mentioned proteins are not the most abundant proteins in the neat plasma, they play a crucial role in regulating immune recognition. This highlights the importance of the “quality” over “quantity” effect in PCs composition on immune cell association [141,142]. The unique composition of PCs is the consequence of interaction between the physicochemical properties of LBNMs and proteins profiles in biological fluid. Thus, it is imperative to know the key functional plasma proteins in PCs and their biological implications and how it would be used in drug delivery. Herein, we selected several key components in PCs (*i.e.*, albumin, immunoglobulins, complement proteins and apolipoproteins) for further discussion.

#### 6.3.1 Albumin

Recent reports have shown that a substantial amount of albumin tends to sediment during the centrifugation for isolating the liposome–PCs complexes. Albumin constitutes the most prevalent protein in blood, interstitial fluid, and lymph, comprising approximately 55 %, 68 %, and 65 % of their total protein, respectively. It is a large protein with a molecular weight of 66.5 KDa synthesized in the liver. Albumin maintains blood homeostasis by buffering pH levels, sustaining oncotic pressure, and transporting endogenous molecules like hormones and long-chain fatty acids [142,143]. Albumin possesses two primary, well-defined hydrophobic binding domains, designated as Sudlow site I and Sudlow site II, which serve as binding sites for various drugs, including warfarin and ibuprofen [144,145].

Furthermore, albumin plays a vital role as a transporter for tumor drug delivery, attributed to several factors [146]. Firstly, the rescue mechanism facilitated by the intracellular neonatal Fc receptor (FcRn) effectively extends the systemic half-life of albumin to approximately 19 days [147]. This prolonged circulation allows for the hitchhiking of therapeutic moieties on albumin, enhancing the pharmacokinetic of LBNMs [148]. Secondly, albumin demonstrates high tumor accumulation due to the EPR effect. Given its abundant presence in blood (40 mg/ml) versus the interstitial fluid (14 mg/ml), serum albumin is transported into tumor tissues via diffusion-mediated flow through leaky vasculature. Additionally, the collapse of lymphatic drainage ensures that albumin remains within the tumor tissues [149]. Thirdly, cancer cells exhibit increased uptake of albumin via macropinocytosis to support their rapid multiplication and growth [150]. Moreover, albumin naturally crosses the vascular barrier by binding to receptor GP60 expressed on endothelial cells and alveolar epithelium [151]. Last, tumor-draining lymph nodes are initiative sites for immune stimulation and lymphatic targeting via albumin-hitchhiking is receiving increasing attention [152]. Collectively, these attributes–the inherent ability to transport hydrophobic molecules, prolonged systemic circulation half-life, infiltration into tumors through leaky vasculature, preferential uptake by cancer cells and lymphatic targeting–make albumin an advantageous protein for anti-cancer drug delivery.

#### 6.3.2 Immunoglobulins

Immunoglobulins are categorized into several classes (*e.g*., IgM, IgD, IgG, IgA, and IgE) and all play an invaluable role in the immune response. Among them, IgG and IgM were identified as the primary immunoglobulins explicitly enriched in the PCs of LBNMs.

IgG represents the most prevalent isotype of immunoglobulins, comprising 10–20 % of plasma proteins. An IgG molecule is structured as a tetramer, consisting of two heterodimers, each heterodimer being composed of a heavy chain and a light chain. These chains are held together by disulfide bonds and non-covalent interactions, resulting in a heterodimer with a molecular weight of approximately 150 kDa. The dimensions of an IgG molecule measure roughly 14.5 × 8.5 × 4.0 nm^3^. IgG can be further classified into four subclasses: IgG1, IgG2, IgG3, and IgG4, which share 90 % amino acid sequence identity but differ in the details of their hinge structure [153]. Each IgG has dual binding activities: to antigen via its amino terminal variable regions and to effector molecules such as FcγRs via its carboxyl terminal constant regions. FcγRs are expressed by many different cell types in the immune system, and their interaction with antibody can initiate a broad spectrum of effector functions that are important in host defense [154]. IgG1 and IgG3 bind FcγRI on macrophages and monocytes with the highest affinity. Some other FcγRs with lower binding affinities with IgG include FcγRIIa (CD32A), FcγRIIb/c (CD32B), FcγRIIIa (CD16A), FcγRIIIb (CD16B), and FcRn [155].

In comparison, IgM is the first to appear during ontogeny and is also the oldest, providing a first line of defense against pathogens. Early IgM antibodies are secreted before B cells have undergone somatic hypermutations and therefore tend to be of low affinity different from IgG. To compensate for the reduced binding efficiency of the monomers, IgM forms oligomers whose multiple antigen binding sites confer high overall avidity. IgM exists in the circulation principally as a pentamer (contain 10 antigen binding sites and the joining (J) chain), and occasionally as a hexamer (containing 12 antigen binding sites without J chain) [156]. In contrast to IgG, the Fc part of IgM is composed of three Ig domains (Cμ2, Cμ3, and Cμ4) and an additional C-terminal tail piece. Each IgM monomer is composed of two heavy chains with five domains (VH, Cμ1, Cμ2, Cμ3, and Cμ4) and two light chains with two domains (VL, CL). It is this multimeric structure that endows IgM with the ability to form strong interactions with several binding partners [157]. IgM seems to be a mushroom-shaped molecule with a central protruding region, which is thought to bind complement component C1q. This complex multimeric structure could explain the 1000-fold greater binding affinity of pentameric IgM to C1q compared with IgG [158]. In addition, hexameric IgM may play an important role in the immune system, since it is up to 20 times more active than pentameric IgM in initiating the complement cascade [159].

IgM antibodies also differ from IgG isotypes due to the relative engagement of effector mechanisms. IgG utilize natural killer cell engagement which can result in antibody-dependent cellular cytotoxicity (ADCC). In contrast, IgM does not bind the Fcγ receptors, and therefore does not exhibit ADCC. However, IgM have very potent complement dependent cytotoxicity (CDC) activity due to the highly avid binding of complement component C1q to IgM. Other effector mechanisms, such as antibody-dependent cellular phagocytosis (ADCP), have also been implicated in the action of IgM [160,161]. PEGylated LBNMs induced the formation of anti-PEG IgM and activated the complement system. Activated C3 fragments (iC3b) led to opsonization and enhanced uptake of PEGylated LBNMs by Kupffer cells in the liver [162,163].

#### 6.3.3 Complement proteins

Another example is the complement system, a critical component of the innate immunity in the blood. The complement system is a network of over thirty different proteins (e.g., C1q, C3, mannose-binding protein, C4b-binding protein, lectin, properdin, factor H and Ficolins) that have been consistently identified in high throughput proteomic screens of PCs [135,164,165]. The complement system can be activated via three different pathways (classical, alternative and lectin pathways) that all converge at the step where the central complement protein C3 is cleaved. Nevertheless, these pathways use different recognition molecules to sense a foreign particle (e.g., antibodies for the classical pathway and mannose binding lectin and ficolin for the lectin pathway), but use similar activation mechanisms to generate enzymes that cleave C3 (known as C3 convertases) [166].

Numerous studies have reported pathway-specific complement activation by LBNMs, with the C1q-mediated classic pathway being a notable example [128]. However, complement activation is a double-edged sword that not only aids the host in defending against pathogens, but also poses the risk of causing damage to host tissues. These species coat the surface of LBNMs, making them susceptible to phagocytosis by macrophages and leukocytes through complement receptors. Activation of the complement cascade further liberates potent anaphylatoxins (C3a, C4a and C5a), that play a crucial role in recruitment and activation of a wide range of inflammatory cells as well as anaphylaxis that subsequently initiate anaphylaxis in some individuals [56,167]. Thus identifying biomarkers that can predict the “risk” of abnormally high complement responders can improve the safety and efficacy of LBNMs. Simberg et al. [168] performed a multifaceted analysis of the factors affecting the complement activation by PEGylated liposomal doxorubicin in plasma. They revealed that plasma concentrations of anti-PEG immunoglobulins (IgG and IgM) exhibited a robust positive correlation with C3 deposition on PLD. Notably, the titers of anti-PEG IgM demonstrated the highest predictive value for assessing the risk of high complement activation by PLD.

#### 6.3.4 Apolipoproteins

Apolipoproteins are essential proteins capable of binding lipids to create lipoproteins, facilitating their efficient transport through the lymphatic and circulatory systems. Additionally, they function as receptor ligands, enzyme cofactors and lipid transfer carriers, regulating lipoprotein metabolism and their absorption into tissues [169]. Apolipoproteins A1 (ApoA1), together with apolipoproteins E (ApoE), was identified by Pattipeiluhu et al. [141] as the most abundant apolipoproteins present on the surface of negatively charged Ambisome-mimicking liposomes.

ApoA1 is a 28 kDa (243 amino acids) protein, constituting 70 % of protein components of high-density lipoproteins. ApoA1 plays a crucial role in the reverse cholesterol transport process, facilitating the transport of excess cholesterol from peripheral cells to the liver. ApoA1 binds to the scavenger receptor B type I (SR-BI), which is predominantly expressed in the liver and steroidogenic tissues, but is also found in monocyte-macrophage and endothelial cells [170].

ApoE, a single-chain glycoprotein composed of 299 amino acids and weighing 34.2 kD, controls cholesterol efflux from tissues to the liver for excretion in conjunction with apoAI. ApoE exhibits a high affinity for binding to the low-density lipoprotein receptor (LDLR) as well as to low density lipoprotein receptor-related protein (LRP), particularly LRP1 and LRP2 [171]. Following intravenous administration, ApoE in the bloodstream binds to LNPs, resulting in the accumulation of LNPs in the liver. The remarkable affinity of patisiran (Onpattro) for the liver was found to be amplified by the adsorption of ApoE onto the surface of LNPs after intravenous administration [24]. The ApoE associated with LNPs serves as an efficient targeting ligand, binding to lipoprotein receptors (e.g., LDLR) on hepatocyte surfaces and thereby inducing endocytosis into the hepatocytes [172].

The concentration of ApoE in the brain ranks second only to that in the liver, primarily synthesized by astrocytes and microglia, with neurons contributing to a lesser extent. LDLR and LRP also mediate the cellular uptake of ApoE in the brain, influences the growth and repair of central nervous system cells. ApoA1 and ApoE can associate with LBNMs to facilitate preferential adsorption onto brain endothelial cells and enhance drug penetration across the blood–brain barrier (BBB) through specific binding to LDLR and SR-B1 [173,174]. Intriguingly, upon contact with ApoE, LNPs exhibit not only the occurrence of protein adsorption on their surface but also a rearrangement of both their surface and core structures. This binding of ApoE results in an elevated cholesterol concentration on the LNP surface, potentially influence LNP endosomal escape and the subsequent release of mRNA [175].

### 6.4 Physicochemical properties of LBNMs on proteins adsorptions

The formation of the PCs around LBNMs can be influenced by numerous factors, notably the physicochemical properties (e.g., size, surface charge, surface property and lipid composition) of the LBNMs. Even minor alterations in the physicochemical attributes of LBNMs can significantly alter their biological effects, potentially leading to a poor correlation between *in vitro* and *in vivo* results. Establishing and understanding the relationship between the physicochemical attributes of LBNMs and their biological interactions poses one of the greatest challenges in the field of bio-nano interactions. The subsequent section specifically focuses on the impact of the physicochemical properties of LBNMs on protein adsorption and its consequences for biological processes.

#### 6.4.1 Size and surface charge

Generally, the size determines total surface area and curvature of the LBNMs, which both affect interaction with the proteins and contribute to the PCs formation. The zeta potential is accountable for the electrostatic interactions with plasms proteins, exhibiting a general trend akin to other nanoparticles, where cationic liposomes typically possess the highest binding capacity, whereas neutral liposomes exhibit the lowest affinity for protein binding [176].

#### 6.4.2 Degradability properties

SM-102 and DLin-MC3-DMA (MC3) are two representative degradable and non-degradable ionizable lipids of LNPs, respectively. MC3 was utilized in the first FDA-approved siRNA therapeutic Onpattro™ while SM-102 served for Moderna’s mRNA-based SARS-CoV-2 vaccine (mRNA-1273). To investigated the differences in mRNA delivery behavior *in vivo* between degradable and non-degradable ionized lipids, Liu et al. [177] encapsulated Cy5-Luc mRNA within DiR-labeled LNPs, utilizing SM-102 and MC3, respectively. By imaging DiR, Cy5, and bioluminescent signals, the LNPs distribution, mRNA release, and mRNA expression can be continuously monitored simultaneously. Compared to MC3 LNPs, the biodegradability of SM-102 helped reduce the tendency of LNPs to transfer from muscle to the liver after intramuscular injection, and the systemic distribution of mRNA was more pronounced regardless of the delivery route.

#### 6.4.3 PEG coating

Conjugating PEG onto the surface of LBNMs, referred to as “PEGylation” is frequently employed to minimize protein binding and prolong the blood circulation of LBNMs. Despite this, the inevitable binding with plasma proteins and the formation of PCs still occurs. Additionally, although PEG has been classified as a “Generally Recognized as Safe” food ingredient by the FDA since 1973 [178], an escalating number of studies indicate that PEG exhibits immunogenic properties in both animals and humans [55,179,180]. Specifically, PEG acts as a hapten, displaying immunogenicity only when conjugated to another substance, such as nanocarriers [181]. Marginal zone B cells are considered the primary immune cells for PEGylated LBNMs. The B-cell receptors (BCRs) of marginal zone B cells recognize PEGylated LBNMs, triggering the secretion of anti-PEG IgM antibodies. The antibody class switching process is facilitated by factors like APRIL (a proliferation-inducing ligand) and BAFF (B cell-activating factor of the tumor necrosis factor family) by B cell helper neutrophils and myeloid cells within the marginal zone [182,183]. The immunogenicity of PEGylated LBNMs significantly impacts their *in vivo* performance, often leading to the accelerated blood clearance (ABC) phenomenon upon repeated administration [184]. This interaction between the induced anti-PEG IgM and subsequent doses of PEGylated therapeutics activates the complement system, facilitating recognition and rapid clearance by the reticuloendothelial system [185].

Furthermore, pre-existing anti-PEG antibodies have been identified in healthy individuals who have not been exposed to PEGylated therapeutic agents [186], and the prevalence of anti-PEG antibodies in the population is rapidly increasing annually [58,187,188]. Even more concerning, the COVID-19 pandemic has not only accelerated the use of LNP-mRNA vaccines but also elevated the levels of anti-PEG antibodies globally [189]. Once anti-PEG antibodies encounter PEGylated LBNMs, they can recognize and bind to the PEG moiety, triggering a series of biological effects, with complement activation being one of the most significant. The activation products, such as C3 fragments, can readily attach to PEGylated LBNMs, making them recognizable by complement receptors and Fc receptors on macrophages, leading to rapid clearance from the systemic circulation [190,191]. Moreover, anaphylatoxins C3a and C5a attach to their specific receptors, C3aR and C5aR, respectively, on innate immune cells like mast cells and platelets, which triggers the secretion of anaphylactic mediators [192]. These inflammatory mediators, including histamine, leukotrienes, platelet-activating factor (PAF), and tryptase, further engage with effector cells, primarily those of the cardiopulmonary system, resulting in the manifestation of hypersensitivity reaction symptoms [193,194]. The membrane attack complex (MAC), alternatively known as the complement activation terminal product sC5b9, has been identified as a mediator that facilitates premature drug leakage from PEGylated LBNMs, ultimately undermining both their efficacy and safety [195,196].

It is noteworthy that most marketed PEGylated drugs utilize PEG with a methoxy terminal group, referred to as methoxy-PEG. In a study by Shimizu et al., the immunogenicity of PEG with different terminal groups was examined, revealing that methoxy-PEGylated liposomes induced higher levels of anti-PEG IgM in mice compared to hydroxyl-PEGylated liposomes [197]. Zhan et al. [198] conducted a comprehensive screening of pre-existing anti-PEG antibodies in large, multicenter clinical serum samples, exploring their binding activity with various PEG materials. Their findings revealed that hydroxyl-PEG can effectively evade binding by these clinical pre-existing anti-PEG antibodies. By substituting methoxy-PEG in commercially available LNP formulations with hydroxyl-PEG, the team observed a significant reduction in complement activation levels in antibody-positive serum, decreased production of anaphylatoxins, and improved plasma stability of LNPs. This modification also led to lower off-target uptake by macrophages, potentially enabling more efficient delivery of LNPs and enhancing the *in vivo* performance of encapsulated drugs. This study uncovered the terminal group selectivity of pre-existing anti-PEG antibodies in the human population during their binding to PEG. It highlights the capability of hydroxyl-PEG to evade recognition by the pre-existing anti-PEG antibodies, which holds immense significance in reducing the adverse effects of LNPs and boosting their efficacy in clinical practice.

#### 6.4.4 Ligands modification

Active targeting drug delivery involves general approach of attaching ligands (e.g., antibodies, peptides or small molecules) to the surface of LBNMs. Despite the presence of cell-specific targeting ligands like monoclonal antibodies, the specificity of active targeting remains a concern. Compared to plain liposomal formulations like Doxil, fewer targeted liposomes have achieved clinical success, including MM-302 (targeting the HER2 receptor with an anti-ErbB2-scFv), 2B3-101 (targeting the glutathione receptor at the blood-brain barrier), and MBP-426 (transferrin-modified liposomes) [[199], [200], [201]]. Furthermore, discrepancies have been observed between *in vitro* and *in vivo* results, often due to the shielding of ligands from their targets by the formation of PCs. An increasing amount of contradictory literature indicates that active targeting strategies may not always lead to increased tumor accumulation, represented by the folic acid (FA)-enabled active drug delivery [[202], [203], [204]].

FA stands out as a stellar molecule often utilized to coat the surface of liposomes and other nanomedicines for active targeting of tumor tissues via recognized the overexpressed FA receptors. Despite numerous research publications and clinical trials, all FA-targeted therapeutic drugs have faced clinical setbacks. Our previous study reexamined the regulatory roles of plasma proteins on FA-functionalized liposomes (FA-sLip) and uncovered a specific interaction between IgM and FA-sLip. The interaction resulted in avid IgM adsorbing onto the surface of FA-sLip and causing several adverse effects, including the loss of binding activity with FA receptor, rapid complement activation, poor pharmacokinetic properties and accelerated blood clearance of FA-sLip [205]. Meanwhile, we verified that discoid-shaped LND instead of spherical liposomes could enhance the *in vivo* performance of FA-targeted LBNMs. LNDs confine the adsorbed IgM to the narrow edge regions, preventing IgM from adopting its antigen-binding conformation, concealing its binding sites for complement protein C1q, and inhibiting its ability to bind complement proteins. Consequently, this approach effectively circumvents the adverse effects of complement activation induced by IgM adsorption, significantly enhancing the *in vivo* performance of FA-targeted drug delivery [128]. Although natural IgM was validated as a key negative plasma protein regulating *in vivo* performance of FA-sLip for the targeted delivery of traditional therapeutic agents, the enhancing immunogenicity can be advantageous for amplifying the immune response of vaccine drugs. Given the unavoidable adsorption of IgM onto FA-sLip *in vivo* and IgM’s diverse roles in immune responses, we proposed a natural IgM-hitchhiking delivery strategy to co-deliver tumor antigens and adjuvants to splenic marginal zone B cells, eliciting lasting antigen-specific antibodies production and cytotoxic T lymphocyte responses *in vivo* for effective tumor immunotherapy [206].

## 7 Summary and prospects

LBNMs represent a transformative drug delivery platform, offering unparalleled opportunities to enhance therapeutic efficacy through their biocompatibility, tunable release profiles, and targeted accumulation. Despite these advantages, clinical translation of LBNMs remains hindered by unpredictable *in vivo* behavior and challenges in establishing robust IVIVC. Although it is generally accepted that *in vitro* dissolution and *in vivo* absorption are correlated, there is still significant scope for the development of a reliable and fully robust model capable of predicting *in vivo* absorption from dissolution profiles. This gap remains due to the variety of factors that influence drug dissolution and absorption. In addition, the conventional IVIVC models is often invalidated upon LBNMs’ exposure to complex biological fluids. This discrepancy arises from the dynamic PCs formation, which dictates LBNMs’ biodistribution, cellular recognition, and immune responses—a complexity often overlooked in conventional IVIVC models.

This review underscores the critical role of PCs in modulating the biological fate of LBNMs, as well as the limitations of conventional IVIVC models that fail to account for dynamic bio-nano interactions. While advancements have improved IVIVC predictability, there are certain questions that warrant immediate attention like; (1) what would be the advancement in dissolution technologies so as to match *in vitro* performance with *in vivo* behavior of LBNMs; (2) to what extent the insights into PCs properties and release kinetics will improve correlation between *in vitro* and *in vivo* fate of LBNMs; (3) is there any possibility of exploiting PC as a tool for modulating release kinetics of LBNMs, *etc*.

Future efforts must prioritize the development of holistic IVIVC frameworks that integrate PC dynamics with dissolution kinetics and physiological models. Some key directions include:

(1) Establishing PCs fingerprint profiles to establish correlations between LBNMs physicochemical properties, key PCs composition, and *in vivo* performance. A typical case involves the above mentioned FA-sLip: FA modification on LBNMs drives the specific adsorption of IgM in the formed PCs, and this IgM-enriched PCs further leads to unexpected alterations in the *in vivo* targeting performance of FA-sLip. This case directly validates the value of PCs fingerprint profiles in linking LBNMs’ surface modification (a physicochemical property), key PCs composition (IgM adsorption), and actual *in vivo* behavior; (2) Expanding PCs analysis to understudied biological milieus (e.g., mucus, cerebrospinal fluid) to refine bio-relevant *in vitro* models; (3) Leveraging AI-driven predictive tools and multi-omics data to decode PCs-mediated biological interactions (4) Advancing bioimaging technologies (e.g., aggregation-caused quenching probes [207,208]). to enable direct real-time visualization and quantification of the LBNMs-PCs complex in both *in vitro* and *in vivo* settings. (5) Additionally, the immunomodulatory effects of PCs—such as complement activation and anti-PEG antibody responses—demand innovative strategies, including terminal PEG modification or biomimetic surface engineering, to enhance clinical safety.

Nanomedicines research must transit from an “empirical design-centric” paradigm to a precision-oriented framework integrating “design-delivery mechanism synergistic research”. The clinical translation bottleneck of LBNMs stems from the complexity of their *in vivo* delivery processes, including dynamic PCs assembly in blood circulation, sequestration by the reticuloendothelial system in liver/spleen, tissue penetration barriers, and intracellular trafficking. Elucidating LBNMs existence forms, immune interactions, and distribution patterns across diverse biological fluids (blood, mucus, interstitial fluid, intracellular fluid) is pivotal for establishing IVIVC models and enabling precision clinical application. Therefore, bridging the IVIVC gap for LBNMs necessitates a paradigm shift toward systems-level understanding, where PCs are not merely passive bystanders but active determinants of therapeutic outcomes. By harmonizing cutting-edge analytical technologies, computational modeling, and mechanistic insights into bio-nano interactions, the field can unlock the full potential of LBNMs, accelerating their transition from bench to bedside.

## CRediT authorship contribution statement

**Huan Wang:** Writing – review & editing, Writing – original draft, Funding acquisition, Conceptualization. **Xiying Wu:** Writing – review & editing. **Junji Wang:** Methodology. **Xiaoyi Wang:** Writing – original draft, Funding acquisition. **Ying Lu:** Supervision, Funding acquisition, Conceptualization.

## Declaration of competing interest

The authors declare that they have no known competing financial interests or personal relationships that could have appeared to influence the work reported in this paper.

## Data Availability

Data will be made available on request.
